# Influence of Assembly Gap Size on the Structure and Properties of SUS301L Stainless Steel Laser Welded Lap Joint

**DOI:** 10.3390/ma14040996

**Published:** 2021-02-20

**Authors:** Hongxiao Wang, Yanxin Wang, Xin Li, Wenquan Wang, Xiwei Yang

**Affiliations:** 1 CRRC Changchun Railway Vehicles Co., Ltd., Changchun 130021, China; wanghongxiao@cccar.com.cn (H.W.); yxwang24@163.com (Y.W.); 2 Key Laboratory of Automobile Materials, School of Materials Science and Engineering, Jilin University, Changchun 130025, China; wwq@jlu.edu.cn (W.W.); yangxiwei@faw.com.cn (X.Y.)

**Keywords:** laser welding, assembly gap, fatigue property, tensile property

## Abstract

The microstructure and properties of laser welding lap joints with different assembly gap sizes are experimentally investigated. The laser weld joint is composed of γ-austenite and δ-ferrite, and the strip ferrite phase is mainly distributed at the austenite grain boundary. The weld metal presents the austenitic-ferritic (AF) solidification mode. When there is a gap between the two plates, a triangular region composed of similar equiaxed crystals can be found, and the size of the cellular crystals in this region decreases significantly. When the assembly gap size increases from 0.1 mm to 0.4 mm, the weld penetration state of the joint changes from full penetration to semi-penetration, and the surface collapse increases. The excessive size of the gap leads to a decrease in the tensile-shear force and fatigue strength of laser welded joints. In order to ensure that the surface morphology and properties of the welded joint meet the quality standard and requirement, the assembly gap should be less than 0.1mm.

## 1. Introduction

The light weight and corrosion resistance of the body are becoming promising directions for both high-speed trains and urban rail vehicles [1]. SUS301L stainless steel (SS) has been applied in the manufacturing of components for automotive and shipbuilding industries for its excellent corrosion resistance and overall performance. In the manufacturing of the body of high-speed trains and urban rail vehicles, the overall weight and strength of the divided side walls determine that the welded joints are of unequal thickness, the commonly used welding method is the resistance spot welding of the stainless steel lap joint, because the welding mechanism is simple and easy to automate in robot production [2]. However, many studies [3,4,5,6] have shown that laser keyhole welding has more advantages in the welding of overlap joints of plants, such as localized heating, small welding deformation, and high penetration depth. Laser keyhole welding has gradually replaced resistance spot welding in automobile and rail vehicle manufacturing. At present, more and more high-speed train and urban rail vehicle manufacturers are adopting laser welding to produce a stainless steel body. Stainless-steel sheets can be connected by laser lap welding, which can minimize the welding deformation with high efficiency. 

It is found that the assembly gap is inevitable in the welding process of the car body, especially in the manufacturing process of the large-size components such as the side walls of the vehicle body. In general, the existence of the assembly gap hinders the energy transfer, reduces the melting rate, and weakens the keyhole effects of laser deep penetration welding. Moreover, laser welding defects are sensitive to the assembly gap size, which have significant impacts on the welding structure, such as leakage welding, virtual welding, cracks, and pores [7]. An excessive gap will lead to quality problems, such as a lack of fusion, poor surface forming, and perforated defects that are harmful to the static strength and fatigue strength of the weld. The quality of welded joints is mainly evaluated by the surface quality and tensile-shear force of weld joints. Therefore, the acceptable range of the assembly gap should be evaluated from a safety perspective [8], and the research on how the gap affects the welding structure and how to evaluate the gaps between adjacent plates has important implications for industrial production. The study of the effect of the gap size on morphologies and mechanical properties of laser overlap welded joints is very important in determining the precision of mechanical pretreatment and the assembly of laser welding panel samples in railway vehicles, the shipbuilding industry, and other heavy engineering industry. A lot of different experimental researches have been done in this area. G. Turichin et al. [9] conducted a study on the laser arc hybrid laser welding of RS E36 steel, indicating that the joint without external defects and the formation were the best when the gap widths were within the range of 0 mm~0.3 mm. Li et al. [10] studied the effect of gap width on the laser-arc hybrid welding(HLAW) of commercial pure titanium, and the research results showed that the surface of welded joints was well formed at the gap width range of 0 mm~0.4 mm. K. Oussaid et al. [11] experimentally investigated the laser welding joint of galvanized steel and reported that the gap width between the plates had a limited effect on the weld quality. Mei et al. [12] found that within the range of an appropriate inter-sheet gap, the variation of the gap size had little effect on the quality of the laser welded joints of galvanized steel, and when the gap was too large or too small, welding defects occurred. 

In this paper, an experimental study on a SUS301L stainless steel laser lap joint is carried out. The effects of the various gap sizes on the microstructures and mechanical properties and fracture modes are researched with gap width sizes from 0 mm to 0.4 mm. 

## 2. Materials and Experimental Methods

SUS301L austenitic stainless steel plates with different thickness were used as the base metal, the dimension of the plates was 150 mm (length) × 50 mm (width), the upper plate thickness was 1.0 mm, and the lower plate was 0.6 mm. The chemical compositions of SUS301L stainless steel provided by the supplier are presented in Table 1. The stainless steel laser lap joints with different assembly gap widths were obtained by adding gaskets with different thicknesses of 0.1 mm, 0.2 mm, 0.3 mm, and 0.4 mm between two plates. The surfaces of the stainless steel plates were polished with silicon carbide waterproof sandpaper from mesh 240 to 600 to remove the greasy dirt and oxides, and then both sides of the welding sample were pressed tightly with a pressing plate and screwed tightly with a self-made clamp. In order to prevent weld deformation, additional steel bars were added to compress the samples. 

A continuous wave (CW) IPG laser (Trudisk 4002) was used with the power adjustment range of 0.4 kW to 4 kW and the light spot diameter of 0.6mm. Through actual production experience and in the literature [13], it is verified that the use of argon or compressed air as the welding protective atmosphere has no obvious effect on weld forming. Therefore, dry compressed air with a flow rate of 30 L/min was used as the sheltering gas from the perspective of cost saving. After welding, the weld was cut along the cross section to prepare metallographic samples. Then, the specimens were ground with silicon carbide waterproof sandpaper from 240 mesh to 2000 mesh, followed by polishing, and etched with the corrosive liquid (5 mL HNO_3_ + 1 mL HF + 44 mL H_2_O). The macrostructure of the welds was measured by optical microscope (OM; ZEISS, Germany). The microstructure and the fracture morphology were measured by the scanning electron microscope (SEM; TESCAN VEGA3) with energy dispersive spectroscopy (EDS; Link-ISIS, Britain). The phase compositions were determined by X-ray diffraction (XRD; D/Max 2500PC, Japan). Electron back-scattered diffraction (EBSD) was used to identify the crystal orientation of the weld. The transmission electron microscope (TEM; JEM-2100F, Japan) was applied to observe the highly magnified microstructure of the weld.

In this work, the laser welding parameters were set as: laser power (LP) 1.45 kW, welding speed (WS) 2.54 m/min, and focusing distance (FD) 0 mm. In the single-factor test, the width of the gap width varied with the thickness of the added gasket. The MTS-810 electro hydraulic-servo testing machine was used for the tensile-shear test of the lap joints, and each tensile-shear load was calculated by the average value of three retests. The fatigue test was performed by the JPL-20E fatigue machine with the cyclic stress ratio (R) at 0.1 at room temperature by the pull-pull loading method. The microhardness test of the welded joint was performed by Vickers hardness tester (MH-3, china) at a load of 200 g and a dwell time of 15 s.

## 3. Results and Discussion

### 3.1. Microstructure and Phase Composition of Laser Welded Joint

**Figure 1:**
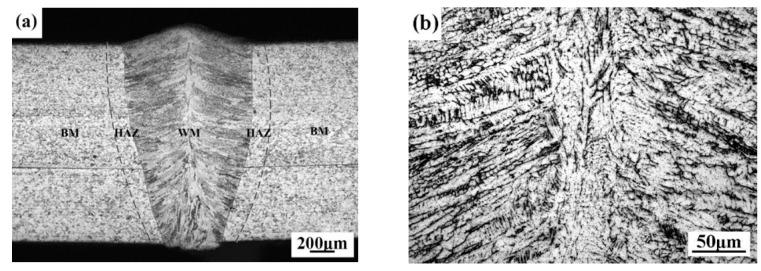

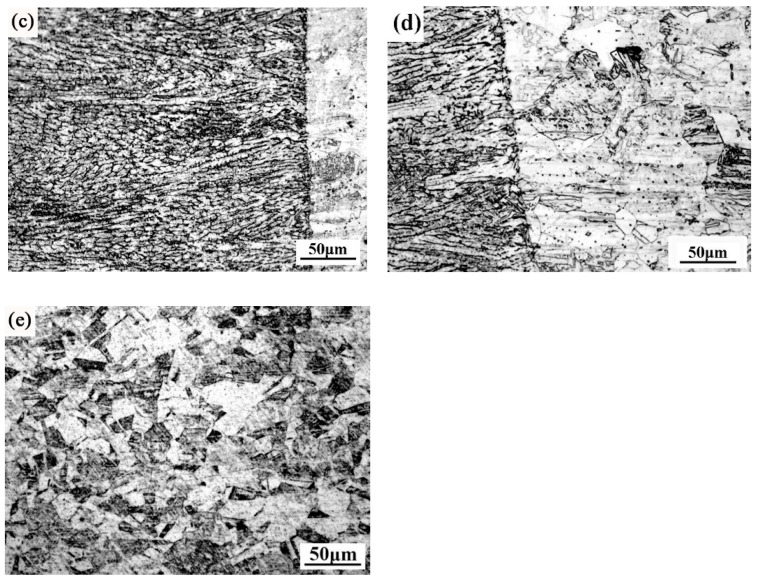
Microstructures of the overlap laser weld: (**a**) cross-sectional appearance; (**b**) weld center; (**c**) weld edge; (**d**) heat affected zone (HAZ); (**e**) base metal.

The typical microstructure of the laser overlap weld with no gap is depicted in Figure 1. As shown in Figure 1a, the weld geometry is “cup-shaped” and the penetration state of the joint is full penetration. The cross-sectional microstructure of the lap joint can be proximately divided into three regions: laser welding zone (LWZ), heat affected zone (HAZ), and base metal (BM). Due to the characteristics of the high energy density and low thermal input [14], the width of the HAZ of the laser welding joint is very narrow (about 0.2 mm). Comparing Figure 1b,e, the microstructure of the base material area is polygonal and the grain size is relatively large, but in the laser welding zone there are columnar crystals and dendrites with different orientations and the grain size is fine. The high magnification morphology of the center of the laser welding zone (Figure 1b) indicates that the crystal solidified microstructure is dendrite and the dendrite microstructures are a larger size in the middle of the weld. The grain size decreases as it gets closer to the edge of the weld, and the crystal solidified microstructure becomes a cellular dendritic crystal and columnar crystal (Figure 1c). The crystals growth orientations are approximately perpendicular to the fusion line. Comparing the microstructures of the HAZ with that of BM (Figure 1d,e), the grain size in the HAZ is obviously increased.

**Figure 2:**
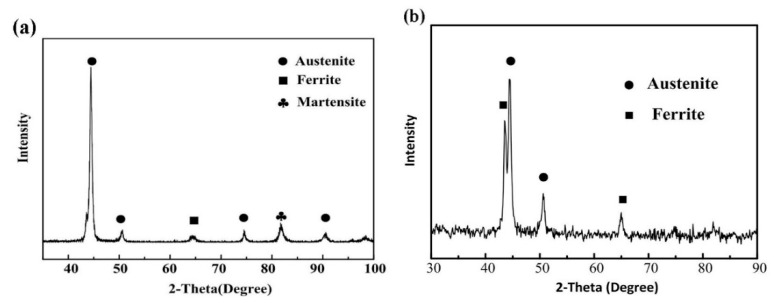
Micro-area X-ray diagrams of (**a**) base metal and (**b**) laser welded joint.

According to the X-ray diffraction analysis results of the base metal and the weld zone (Figure 2), the base metal is mainly composed of austenite phase (PDF#52-0513) and a small amount of ferrite (PDF#85-1410) and martensite. The laser welding zone is composed of austenite phase and ferrite, and no martensite was detected after laser welding. This is mainly due to the martensite being decomposed by the laser high temperature heating, and the stability of austenite and ferrite was increased because of alloying elements during the cooling process. Besides, there is no cold deformation treatment after laser welding, so martensite disappears in the LWZ. 

**Figure 3:**
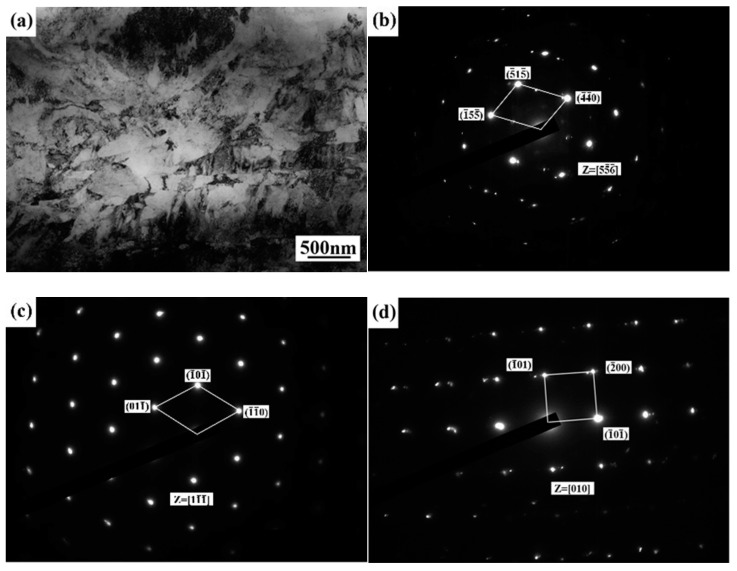
Transmission electron microscope (TEM) image of the base metal: (**a**) TEM bright field image of the base metal; (**b**) SAED pattern of austenite; (**c**) SAED pattern of ferrite; (**d**) SAED pattern of martensite.

Figure 3 illustrates the transmission electron microscope analysis results of the base metal. Figure 3a shows the TEM bright field image of the base metal, which is composed of white block structures and black flake structures distributed along the rolling direction and attached to the white structure grain boundary. According to the selected area electron diffraction (SAED) results, the ferrite phase in the base material of stainless steel is mainly precipitated at the austenite grain boundary. The SAED results further prove that the base metal of stainless steel is mainly made up with the austenitic phase and a small amount of ferrite and martensite, as shown in Figure 3b–d.

**Figure 4:**
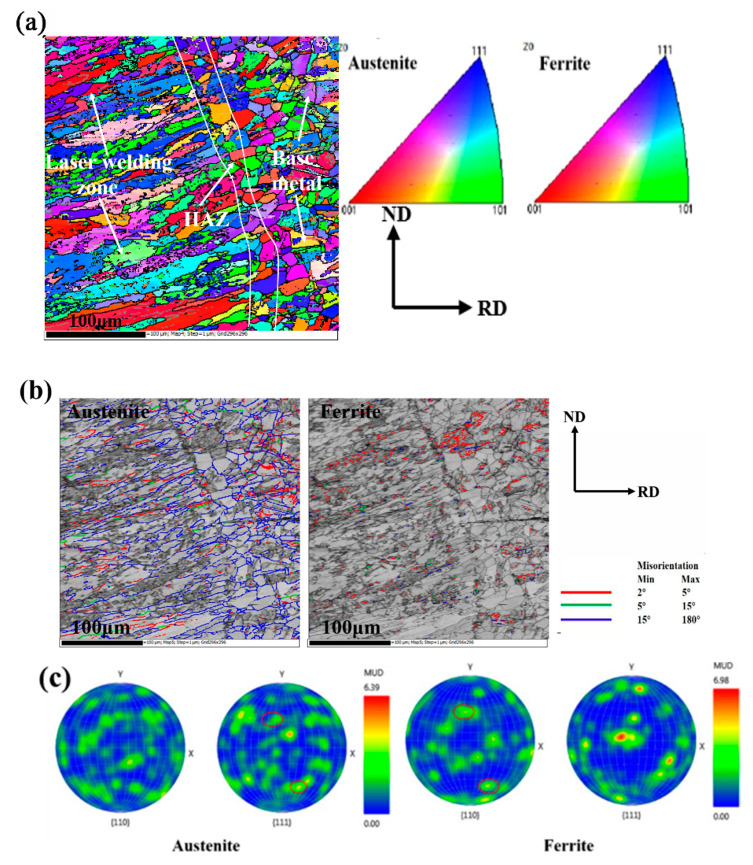
Electron back-scattered diffraction (EBSD) maps of the microstructure along the fusion boundary in the transverse weld: (**a**) orientation map and inverse pole map; (**b**) grain boundaries of (**a**); (**c**) pole figures of the close-packed plane.

Figure 4a is the EBSD image of the stainless steel laser weld. The grains were colored according to the crystallographic orientation. As shown in Figure 4a, the laser welding seam is mainly composed of austenite (red grains) and ferrite (blue grains). The crystal morphology of the base metal is an equiaxed grain structure and the grains of various colors are uniformly distributed in the base metal, so the base metal has no obvious preferred orientation. The grain orientation in the HAZ is the same as that of the base metal, but the grain size increases due to recrystallization. The shape of grains in the base metal and the HAZ is a polygon and the grains in LWZ changes to the shape of the skeleton with a certain direction. Figure 4b shows the grain boundary distribution of austenite and ferrite in the welded joint. It can be seen that ferrite is mainly distributed at a small angle grain boundary, while austenite is distributed at a large angle grain boundary. The polar diagram figures of the closed-packed planes of austenite and ferrite are shown in Figure 4c, and the red circles mark the closed-packed planes. As can be seen, the {111} closed-packed plane of austenite is parallel to the {110} closed-packed plane of ferrite in the laser weld. It can be found that the austenite and ferrite in the weld had a specific orientation relationship (K-S relationship).

**Figure 5:**
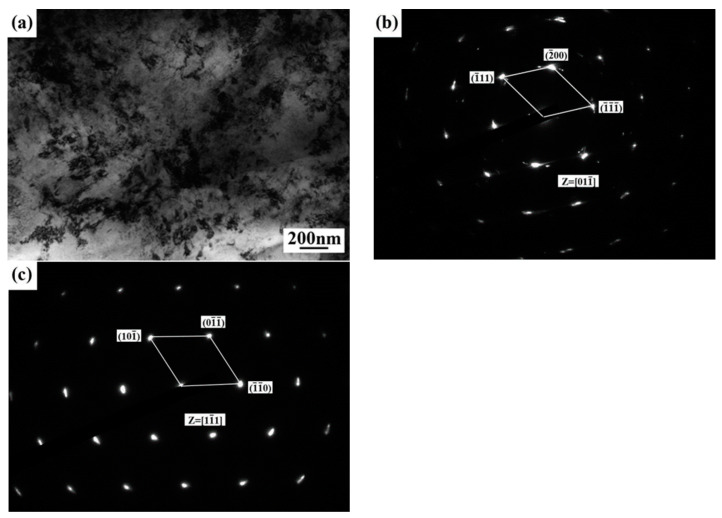
TEM image of weld: (**a**) TEM bright field image of weld; (**b**) SAED pattern of austenite; (**c**) SAED pattern of ferrite.

Figure 5 illustrate the TEM image of the laser weld. Figure 5a is the bright field image of the welded joint, showing that the ferrite phase in weld was precipitated mainly at the austenite grain boundary, and the ferrite phase was precipitated inside the austenite grain. The results further prove that austenite and ferrite present a K-S relationship: (011)_δ_//(111)_γ_, [111]_δ_//[011]_γ_.

According to the chemical composition of SUS301L stainless steel and the pseudo-binary phase diagram of Fe–Cr–Ni [15], the microstructure of the base metal should be a mixture of austenite and ferrite. The existence of martensite in the rolled base metal is due to the austenite matrix producing dislocation, stratification, and a stratification beam after cold deformation, and a large number of parallel and interlacing shear bands are formed by the accumulation of the deformation. The strain-induced martensite was nucleated at the intersections of these shear zones, and even in a single shear zone, and distributed on the austenite matrix, which had a certain strengthening effect on the matrix. With the increase of the deformation, martensite nucleates and grows, and finally joins together to form lathy martensite.

The solidification and solid phase transition of austenitic stainless steel laser weld metal can be deduced by combining the Scheffler diagram and the pseudo-binary sections of the Fe-Cr-Ni ternary system [16]. At the same time, the composition segregation in the process of non-equilibrium solidification at a rapid cooling rate also has an effect on the solidification mode and microstructure of the weld [17]. In this study, if the burning loss of elements during laser welding is ignored, the values of the equivalent contents (wt %) of Cr_eq_ and Ni_eq_. could be calculated from the compositions of the base metal and the solidification mode and microstructure could be confirmed by the values. The calculation formulas are as follows [18]:

Cr_ep_=%Cr+%Mo+1.5%Si+0.5%Nb (1)

Ni_ep_=%Ni+30%C +0.5%Mn. (2)

According to the calculation results, the Cr_eq_ and Ni_eq_ in the laser weld joint is about 18.5% and 8.9%, respectively. Accordingly, the Cr_eq_/Ni_eq_ ratio is about 2.08, the primary phase of the weld during equilibrium solidification should be the δ- ferrite phase (F mode). However, because the cooling rate and solidification rate of the laser welding weld are very fast, the solidification mode of the laser weld should be the austenitic (A) or austenitic-ferritic (AF) mode at the condition of non-equilibrium solidification. The research results of the microstructures of the weld have shown that the laser weld is composed of austenite and ferrite phases, so the weld metal solidification process exhibits the austenitic-ferritic (AF) solidification mode. That is, austenite was first formed during solidification, and some ferrite was formed through the eutectic reaction before the end of solidification. The AF solidification pattern occurs mainly because sufficient ferrite forming elements (Cr and Mo) are concentrated at the sub-grain boundary during solidification to induce the formation of ferrite. The solidification reaction can be expressed as L→L+A→L+A+(F+A) _eutectic_→A+F. The ferrite in the weld after solidification exists in the cellular crystal boundary and the dendrite boundary that is consistent with the TEM analysis results.

### 3.2. Influence of Assembly Gap Size on the Morphologic Observation of Joint

#### 3.2.1. Influence of Assembly Gap Size on the Surface Appearance and Cross-Sectional Macro Morphology of Joint

**Figure 6:**
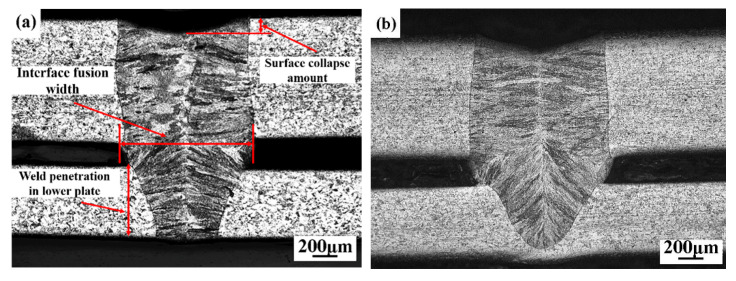

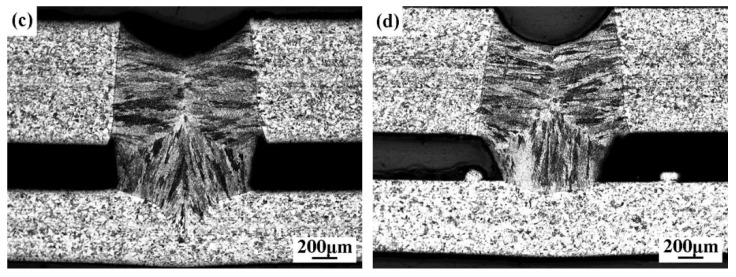
The cross-sectional macro morphologies of welds with different gap widths: (**a**) 0.1 mm; (**b**) 0.2 mm; (**c**) 0.3 mm; (**d**) 0.4 mm.

The cross-sectional macro morphologies of the weld joints with different assembly gap sizes are shown in Figure 6. The welding quality is determined by the penetration depth, the weld width, the forming quality of the weld surface, and so on [19]. The surface collapse amount, interface fusion width, and weld penetration in the lower plate are marked in Figure 6a. With the increase of the gap size from 0.1 mm to 0.4 mm, the penetration state of the joint changes from full penetration to semi-penetration, and the surface collapse occurs, which is due to the lack of filler weld metal caused by too large gap size (Figure 6b). Figure 6c,d shows that the surface collapses become more serious. In addition, the growth of cellular crystals of the weld in the lower plate changes from lateral growth to upward growth with the increase of the assembly gap size. 

**Figure 7:**
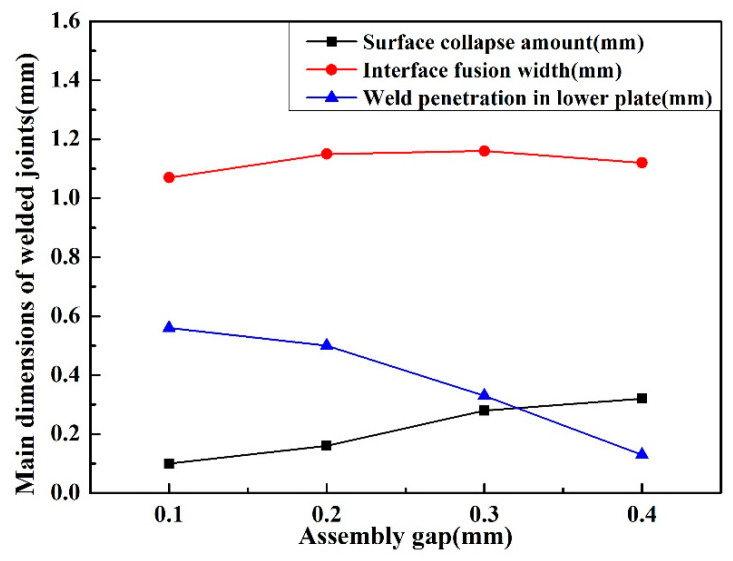
The effect of the assembly gap on the geometric parameters of the weld metal.

Figure 7 shows the curves of the interface fusion width, weld penetration depth in the lower plate, and the surface collapse amount with the the change of gaps in the same welding parameters. When the gap increased from 0.1 to 0.4 mm, the weld penetration depth in the lower plate decreased from 0.58 mm to 0.13 mm. This was mainly due to the loss of laser energy at the gap with the increase of the gap, and the excavation effect of the metal vapor generated by the laser radiation at the bottom of the hole was weakened, leading to the decline of the weld penetration depth. It is found that the size of gap has little effect on the interface fusion width. It could be deduced that as more liquid metal flows into the gap and melts the side wall, the loss of plasma in the gap results in the decrease of the plasma absorption on the laser, showing that the weld width is no different. With the increase of the gap from 0.1 mm to 0.4 mm, the surface collapse amounts are 0.1 mm, 0.16 mm, 0.28 mm, and 0.32 mm, respectively. The weld metal is not enough to fill the gap because of the excessive gap width, leading to a gradual increase of the collapse amount of the weld surface. According to the PRC National Standard GB/T 22085.1 of the electron and laser-welded joints guidance on quality levels for imperfections [20], the surface collapse amount of a weld should not exceed 10% of the upper plate thickness (0.1 mm), so the assembly gap should be controlled less than 0.1mm in order to ensure the surface quality of welded joint when complying with the PRC National Standard.

#### 3.2.2. Microstructure Analysis of Typical Defective Weld Joints

**Figure 8:**
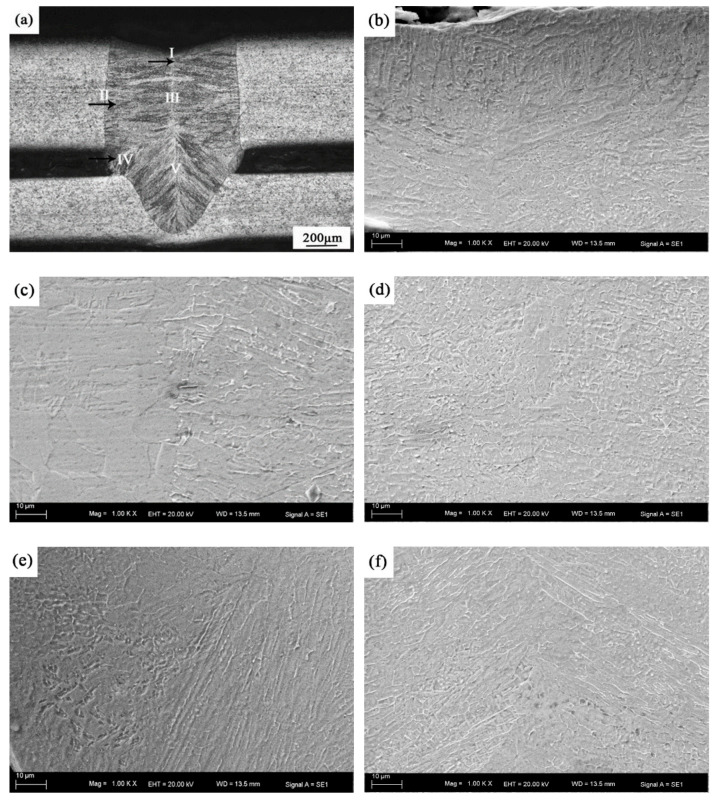
Microstructure of various regions of welded joint with 0.2mm gap: (**a**) optical morphology of the cross section of the weld; The magnified morphologies of region (**b**) Ⅰ, (**c**) Ⅱ, (**d**) Ⅲ, (**e**) Ⅳ and (**f**) Ⅴ.

In order to study the influence of the assembly gap on the microstructure of the weld, the cross-sectional microstructures of the joint with a gap of 0.2 mm is shown in Figure 8. In Figure 8a, the surface of the weld is severely collapsed because of the gap being too large to lead to insufficient filler metal. The microstructure of the LWZ has some differences from that of a joint without a gap. Five regions were characterized in the LWZ according to the different crystal microstructures and orientations, marked as region Ⅰ to Ⅴ, as seen in Figure 8a. The cooling rate has an effect on the crystal morphology and direction, and the faster cooling rate promoted the formation of columnar crystals, while the general cooling rate promoted the formation of dendrites [21]. Figure 8b is the magnified morphology of region I, there is a thin layer of the columnar crystal structure that crystallizes from the outside to the inside along the direction of the thermal diffusion at the surface of the weld because that is where the cooling rate is the fastest. At the middle of laser weld zone (Figure 8c,d), the grains preferentially form dendrites, and the direction of crystallization is from the edge of the weld to the center. This is because the heat diffuses from the weld center to the base metal on both sides in region I and Ⅱ, and the degree of supercooling is relatively gentle. Suutala [22] believed that the cooling rate of the molten pool has an effect on ferrite morphology. Figure 8e is the magnified morphology of region Ⅳ, it can be seen that some fine columnar dendrites show obvious directionality because of the rapid cooling rate caused by the gap. When there is a gap between the two plates, the condition of the molten pool metal growing perpendicular to the fusion surface was destroyed, and a triangular region composed of similar equiaxed crystals can be found, which is composed of three points: the junction point of the two plate gaps with the upper plate fusion line and the weld metal, the junction point of the two plate gaps with the lower plate fusion line and the weld metal, and the junction point of the crystallization growth line of the upper and lower weld metal (Figure 8e). The size of the cellular crystals in region Ⅳ decreased significantly. In region Ⅴ (Figure 8f), the columnar grains were radially distributed from the edge of the weld to the center.

### 3.3. Influence of Assembly Gap Size on the Properties of Joints

#### 3.3.1. Influence of Assembly Gap Size on Tensile-Shear Force of Joints

**Figure 9:**
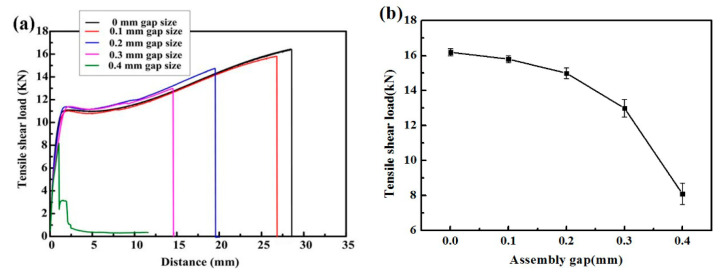
The effect of assembly gap size on the joint tensile and shear forces; (**a**) tensile-shear force-displacement curve; (**b**) tensile-shear force-assembly gap curve.

Figure 9 shows the effect of the assembly gap size on the joint tensile and shear forces. As shown in Figure 9a, when the assembly gap is less than 0.3 mm, the tensile specimens are in a continuous transition during the tensile test, showing relatively obvious elastic and plastic stages. In the elastic stage, the four curves with a gap of 0–0.3 mm almost coincide, indicating that the gap size has no significant effect on the tensile-shear force of the weld when the gap is less than 0.3 mm. However, the curves separate and the fracture displacement changes obviously in the plastic stage. When the assembly gap is 0.4 mm, a step can be seen in the curve, which is due to the influence of the torque in the process of drawing and shearing, due to the large gap size. From Figure 9b it can be seen that the tensile-shear force of stainless steel weld joints decreased with the increase of the assembly gap size. The existence of the assembly gap intensifies the stress concentration and produces torsion and results in the decline of the mechanical properties of the joints. With the gap increasing from 0 mm to 0.4 mm, the minimum tensile-shear load of the joints decreased from 16.2 kN to 8.1 kN. The weld near the gap is more likely to cause stress concentration, which greatly worsens the mechanical properties of the weld. The above results indicate that the excessive size of the gap leads to the reduced tensile-shear force of the welded joints. The quality standard of the rail vehicle production enterprise requires that the tensile-shear force of the joint should not be less than 11 kN, so the assembly gap should be kept less than 0.3 mm in view of the tensile and shear performance of the weld. 

The tensile fracture of the lap joint is the result of the joint action of tensile stress and shear stress. There are two kinds of shear fracture modes for stainless steel laser welds: the heat affected zone fracture and interface fracture [23,24]. In Figure 9a, the black curve shows the fracture curve of specimen with no gap fracturing at the HAZ of the joint. The fracture mode of the joint is related to the microstructure and the hardness of the base metal and weld. According to the results of hardness testing, the average microhardness of the base metal is about 300 HV, and the average microhardness of the laser welding zone is about 240 HV. Because the microhardness of the cold-rolled plate is greater than that of the weld, the tensile and bending deformation are concentrated near the HAZ. The grains in the HAZ become coarse because the HAZ is kept at a high temperature for a long time, and it is the weakest area of the whole joint. Eventually it breaks along the fusion line—the HAZ fracture. The green tensile shear force-displacement curve in Figure 9a shows the specimen with a 0.4 mm gap fracturing at the interface of the welded joint. When the gap increases to 0.4 mm, the tensile-shear force of the joint decreases to 8.1 kN, which is 50% lower than that of the non-gap joint. The fracture mode changes from the fracture in the HAZ to the fracture in the interface. This is mainly due to the large assembly gap causing the torque generated in the tensile process and the increase of the non-uniformity of the welded joint strength.

**Figure 10:**
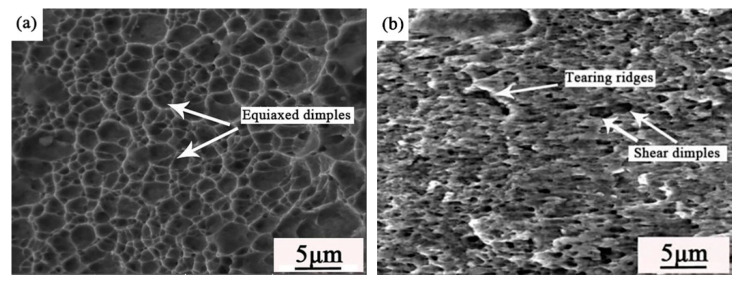
Fracture surface morphologies of joints with gap sizes of (**a**) 0 mm and (**b**) 0.4 mm.

The fracture surface morphologies of laser welded joints with gap widths of 0 mm and 0.4 mm are illustrated in Figure 10. Figure 10a shows the HAZ fracture surface morphologies of the weld with no gap. It can be seen that some equiaxed dimples vary in size formed under tensile normal stress exhibits obviously tensile fracture characteristics. The larger the dimple size, the better the plasticity it is. When the welded joint breaks at the HAZ, it shows that the bearing capacity of the weld is strong enough to meet the production. Figure 10b shows the interface fracture surface morphologies of the welded joint with a 0.4 mm gap, which shows obvious characteristics of the shear fracture. The dimples elongate to form obvious shear lips and tearing ridges by the shear stress. When the welded joint breaks at the interface, it shows that the bearing capacity of the weld is insufficient. Therefore, the interfacial fracture mode should be avoided as much as possible.

#### 3.3.2. Influence of Assembly Gap Size on the Fatigue Performance of Joints

**Figure 11:**
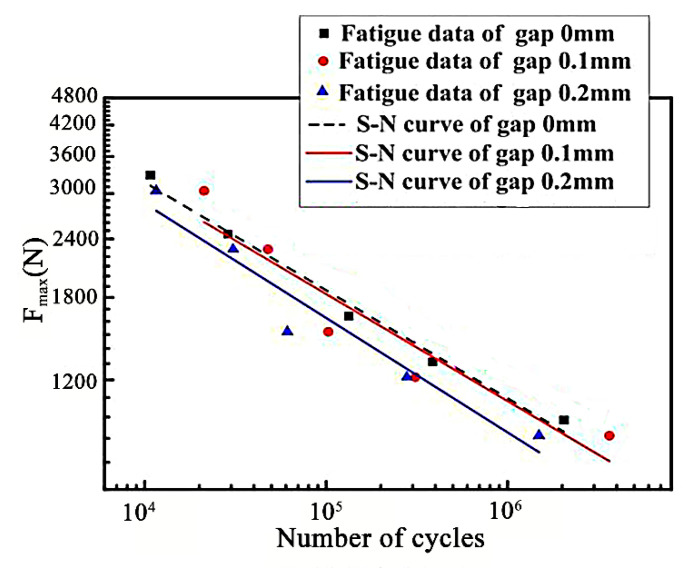
Fatigue curves of the different assembly gap.

The fatigue strength of stainless steel laser welded joints is an important property that affects the performance of the stainless steel railway vehicle. In the manufacturing process of the side wall structure of the railway vehicle body, the frame and skin are connected by overlapped joints. The overlapped weld zones have obvious stress concentration under the action of cyclic loading. The other main factors causing stress concentration is the assembly gap, which will increase the non-uniformity of welded joint strength and lead to the decrease of the fatigue performance of welded structures. The above study shows that when the assembly gap reached 0.3 mm or more, the laser welding joints did not meet the quality standards because the surface collapse amount of the weld exceeded 0.1 mm and the tensile-shear force of the weld was less than 11 kN. Therefore, only the fatigue properties of joints with gap sizes less than 0.2 need to be studied. The fatigue performance of joints was tested at room temperature with a cyclic stress ratio r = 0.1 and pull-pull loading. The tensile and shear fatigue load without cracking during 10^7^ cycles is defined as the conditional fatigue load. Figure 11 shows the fatigue curve drawn by the maximum tension shear load (F_max_) and cycle number (N_f_). As shown in Figure 11, the assembly gap has a certain impact on fatigue performance. When the assembly gap sizes increased from 0 mm to 0.2 mm, the fatigue limit of the weld joints decreased from 822 N to 760 N. Based on the least squares method, specimen with a 0 mm gap illustrate a higher fatigue performance compared with specimens with a 0.2 mm gap but illustrate a similar fatigue performance compared with specimens with a 0.1 mm gap. Therefore, the assembly gap should be controlled less than 0.1 mm in order to ensure the fatigue performance of the joint. 

**Figure 12:**
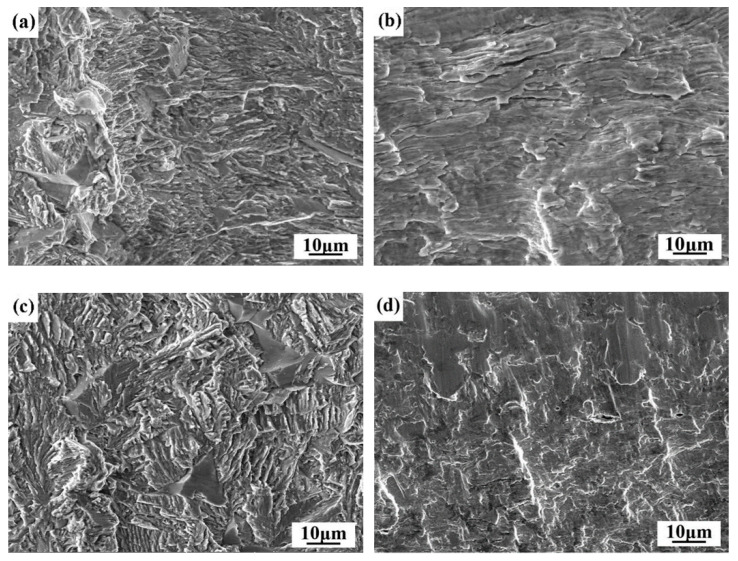
SEM morphologies of the fatigue fractures: (**a**) fatigue crack propagation zone and (**c**) final fracture zone of specimen with a 0 mm gap; (**b**) fatigue crack propagation zone and (**d**) final fracture zone of specimen with a 0.2 mm gap.

The failure positions of the welded joints with an assembly gap from 0 to 0.2 mm are all at the edge of the joints on the lower plates. Figure 12 shows the fatigue fractography of laser welded joints with a 0 mm and 0.2 mm gap. As shown in Figure 12a,c, the surfaces of the fatigue crack propagation zones are rough, and the width of the fatigue striation of the specimen with a 0 mm gap was larger than that of the specimen with a 0.2 mm gap, indicating that the growth rate of the fatigue crack was faster. The morphology of the final fracture zone of the specimen with a 0 mm gap was rough and formed an obvious shear lip, showing obvious plastic fracture characteristics (Figure 12b). The morphology of the final fracture zone of the specimen with a 0.2 mm gap was crystalline, showing obvious brittle fracture characteristics (Figure 12d).

## 4. Conclusions

(1) The microstructure of a SUS301L stainless steel laser welded joint consists of austenite and strip ferrite, and the ferrite phase mainly is precipitated at the austenite grain boundary. The ferrite and austenite phases in the weld present the K-S relationship. The weld metal solidification mode is called AF, which means austenite formed firstly during solidification, and some ferrite was formed in the cellular crystal boundary and the dendrite boundary through eutectic reaction before the end of solidification.
(2) The assembly gap has obvious influence on the morphology of the welded joints. With the increase of the gap size from 0.1 mm to 0.4 mm, the weld penetration state of the joint changes from full penetration to semi-penetration, and the surface subsidence occurs, which is due to the lack of filler metal caused by a too large gap size, and the growth of the cellular crystals changes from lateral growth to upward growth from the lower plate. When there is a gap between the two plates, a triangular region composed of similar equiaxed crystals can be found, and the size of the cellular crystals in this region decreases significantly.
(3) The excessive size of the gap will lead to a decrease in the tensile-shear force and fatigue strength of joints. With the gap size increasing from 0 mm to 0.4 mm, the minimum tensile-shear load of the joints decreased from 16.2 kN to 8.1 kN and the fracture mode changed from the heat-affected fracture (shear fracture) to the interfacial fracture. The assembly gap had a certain impact on fatigue performance. In order to ensure that the surface morphology and mechanical properties of the welded joint can meet the quality standards and requirements, the assembly gap should be less than 0.1 mm.

## Figures and Tables

**Table 1:** Chemical compositions of SUS301L austenitic stainless steel (wt%).

C	Si	Mn	P	S	Ni	Cr	N	Fe
0.03	1.00	2.00	0.045	0.030	7.00	17.00	0.10	Bal.

## Data Availability

The data presented in this study are available on request from the corresponding author.
